# A Novel *Lens orientalis* Resistance Source to the Recently Evolved Highly Aggressive Australian *Ascochyta lentis* Isolates

**DOI:** 10.3389/fpls.2017.01038

**Published:** 2017-06-14

**Authors:** Rama H. R. Dadu, Rebecca Ford, Prabhakaran Sambasivam, Dorin Gupta

**Affiliations:** ^1^Faculty of Veterinary and Agricultural Sciences, University of MelbourneDookie, VIC, Australia; ^2^Environmental Futures Research Institute, School of Natural Sciences, Griffith UniversityNathan, QLD, Australia

**Keywords:** ascochyta blight, *Ascochyta lentis*, lentil, wild lentils, screening

## Abstract

Substantial yield losses and poor seed quality are frequently associated with Ascochyta blight infection of lentil caused by *Ascochyta lentis*. Recently reported changes in aggressiveness of *A. lentis* have led to decreased resistance within cultivars, such as Northfield and Nipper in Australia. Furthermore, the narrow genetic base of the current breeding program remains a risk for further selective pathogen evolution to overcome other currently used resistances. Therefore, incorporation of potentially novel and diverse resistance genes into the advanced lines will aid to improve cultivar stability. To identify these, 30 genotypes sourced from five wild species (*Lens orientalis, L. odomensis, L. ervoides, L. nigricans* and *L. lamottei*), including eight previously reported resistance sources, were screened for disease reaction to two recently isolated and highly aggressive isolates. Subsequently, two *L. orientalis* accessions were found highly resistant and a further six *L. nigricans*, one *L. odomensis*, one *L. ervoides*, one *L. lamottei*, and one *L. orientalis* accessions were moderately resistant. Several of these were more resistant than the currently deployed resistance source, ILL 7537. Furthermore, *L. orientalis* accession ILWL 180 was consistently resistant against other highly aggressive isolates recovered from diverse geographical lentil growing regions and host genotypes, suggesting stability and potential for future use of this accession in the Australian lentil breeding program.

## Introduction

Lentil (*Lens culinaris* Medikus ssp. *culinaris*) (2n = 14), a cool season high protein (26%) food legume cultivated around the world, is ranked fifth in size of global production among legumes at 4.88 million tons (mt) (FAOSTAT, 2014). However, a significant reduction in lentil productivity (30%) was reported during 2013–2014 in Australia (FAOSTAT, 2014), largely due to the disease ascochyta blight, caused by necrotrophic fungus *Ascochyta lentis (A. lentis)*. This disease is of global concern (Kaiser and Hannan, 1986; Erskine et al., 1994; Nasir and Bretag, 1997a; Muehlbauer and Chen, 2007), reducing yields and seed quality (Morrall and Sheppard, 1981; Gossen and Morrall, 1983). It causes an estimated $15.3 million AUD in losses to the Australian lentil industry alone due to reduced production and disease management costs (Murray and Brennan, 2012).

To date, integrated disease management approaches combining best cultivation practices, application of fungicides and cultivars with moderately resistant or resistant ratings have sustained the industry in the presence of *A. lentis* (Hawthorne et al., 2012). However, continuous cultivation of relatively few resistant cultivars with narrow genetic base has likely led to episodes of resistance breakdown through selection of adapted and aggressive isolates (Nasir and Bretag, 1997b; Davidson et al., 2016; Sambasivam et al., 2017). This has also occurred for several Canadian cultivars including Laird (Ahmed and Morrall, 1996) and breeding line ILL 5588 (Tullu et al., 2010). ILL 5588 was also introduced into Australia after its success in Canada and Northfield, a selection from ILL 5588 (Ali, 1995) along with Indianhead were employed either individually or in combination to breed resistant cultivars. However, an increased susceptibility of Northfield to the Australian *A. lentis* population was detected within six seasons after its commercialization (Nasir and Bretag, 1997b). Consequently, this most likely led to the demise of the new Australian cultivar Nipper after just four seasons though carrying an Indianhead pedigree, which is still resistant to major Australian isolates (Davidson et al., 2016). Meanwhile, other Australian cultivars, such as PBA Ace, PBA Blitz, PBA Bolt, PBA Jumbo, PBA Jumbo2, PBA Herald XT, and PBA Hurricane XT, were developed containing a CDC Matador pedigree with *A. lentis* resistance from Indianhead (Pulse Australia, 2016). Several of these were found susceptible or moderately susceptible to recently detected highly aggressive Australian isolates, with predicted increasing industry reliance on those that remained somewhat resistant, such as PBA Jumbo2 and PBA Harricane XT (Davidson et al., 2016). This will again likely lead to increased selection pressure on the highly variable pathogen population (Nasir and Bretag, 1997b, 1998; Davidson et al., 2016; Sambasivam et al., 2017), to evolve and overcome the relatively few resistance sources upon which the industry is currently reliant.

Therefore, a major goal for the Australian lentil breeding program remains to introgress novel resistance genes/alleles or combinations thereof to improve the stability and further enhance durability of resistance to *A. lentis* within elite cultivated backgrounds. Several previous investigations have uncovered sources for novel *A. lentis* resistance in all five wild relative *Lens* taxa (*L. orientalis, L. odomensis, L. ervoides, L. nigricans*, and *L. lamottei*) (Bayaa et al., 1994; Ahmad et al., 1997; Tullu et al., 2010). Although crossing incompatibility exists among such broad germplasm (Ladizinsky, 1979; Ladizinsky et al., 1984), inter-specific fertile hybrids were produced through conventional techniques between accessions of *L. culinaris* and *L. orientalis* within the primary gene pool (Ladizinsky, 1999; Fratini et al., 2004; Gupta and Sharma, 2007). Success was achieved with the aid of GA3 application and embryo rescue techniques for the more incompatible crosses (Cohen et al., 1984; Ahmad et al., 1995; Tullu et al., 2013). Subsequently, segregating populations for ascochyta resistance were successfully produced from *L. culinaris* × *L. orientalis* and *L. culinaris* × *L. ervoides* crosses, within which resistance was simply inherited (Ahmad et al., 1997). More recently, Fiala et al. (2009) successfully transferred anthracnose resistance from *L. ervoides* to *L. culinaris* and developed RIL population which was later evaluated by Vail (2010). This cross was also used to generate backcrosses which were reported to be stable and without any phenotypic linkage drag with yield. Selected breeding lines evaluated under field conditions were reported to be highly resistant to anthracnose under high disease pressure.

The hypothesis is that the wild species of lentil possess novel and diverse resistance alleles/genes to ascochyta blight and the resistance conferred is potentially durable. Therefore, the aims of the current study were to 1) uncover potentially novel wide germplasm sources of resistance to the most aggressive isolates of *A. lentis* recently detected in Australia and 2) determine potential stability of the resistance(s) through screening against a diverse collection of isolates from the current population.

## Materials and methods

### Plant and fungal materials

Thirty wild lentil accessions were provided by the Australian Grain Gene bank (AGG), Horsham, Victoria (**Table 3**). Two cultivars routinely used to discriminate the reaction of *A. lentis* (Davidson et al., 2016; Sambasivam et al., 2017), ILL 6002 (susceptible) and ILL 7537 (resistant) were included as controls. Three seeds per genotype were sown in 10 cm pots filled with pine bark potting mix, fertilized with Nitrosol, Amsgrow® (4.5 mL/L) on a weekly basis and watered on every alternative day. Three replications (three inoculated and three non-inoculated/control pots) were included per each treatment combination (genotype × isolate) After sowing, pots were placed in a glass house at the Dookie Campus, University of Melbourne, Victoria maintained at 20 ± 5°C under 16/8 h day/night photoperiod until inoculation. Considering germ inhibition in wilds, 21 day old seedlings were used for bioassay, such that the leaf number and number of nodes were a minimum of 8–10 and a minimum of 4, respectively, in both wilds and 14 day old cultivars. Post inoculation, pots were moved into a Conviron growth cabinet replicating glass house conditions.

Single spore cultures of four highly aggressive isolates (FT13037, FT13038, FT13050, and FT13027) and one low aggressive isolate (F13082) of *A. lentis* were obtained from the South Australian Research and Development Institute (SARDI) (Table 1). These were sub cultured on the 8th day from wild lentil genotypes sowing on potato dextrose agar media (PDA) plates and incubated for 14 days at 22°C, 12/12 h dark/light cycle under florescent (OSRAM TLD/18W) and near Ultra Violet (UV) lights (PHILIPS BLB/18W).

**Table 1:** Details of *A. lentis* isolates used in the study.

**Isolate**	**Nature of isolate**	**Lentil–cultivar**	**Location in Australia**	**Date collected**
FT 13027	Aggressive	Blitz	Maitland, South Australia (SA)	2/08/2013
FT 13037	Aggressive	Flash	Urania, SA	29/07/2013
FT 13038	Aggressive	Cumra	Urania, SA	29/07/2013
FT 13050	Aggressive	Nipper	Mallala, SA	30/08/2013
F13082	Non-aggressive	Nipper	Pinery, SA	24/09/2013

### Experimental design

Preliminary bioassays were conducted to reconfirm the aggressiveness of the two isolates (FT13037 and FT13038) by screening them against three host differentials with known resistance levels comprising ILL 7537 (resistant), Nipper (moderately resistant-moderately susceptible) and ILL 6002 (susceptible) (Davidson et al., 2016; Sambasivam et al., 2017).

Later, experiments were carried out in two stages. Initially, all 30 genotypes were screened against isolates FT13037 and FT13038 to determine disease responses and identify those with lowest disease severity. Subsequently, the highly resistant genotype (ILWL 180) identified was assessed for its reaction to all five isolates. All the experiments were set out in a completely randomized design under controlled conditions with 3 replications. Initial screening included 120 treatment combinations (30 genotypes × 2 isolates × 2 inoculation treatments (inoculated or non-inoculated)), whereas stability experiments included 10 treatment combinations (1 genotype × 5 isolates × 2 inoculation treatments (inoculated or non-inoculated)).

### Preparation of inoculum and bioassay

Preparation of spore suspension and subsequent inoculation of pots was followed as described in previous studies (Ford et al., 1999; Davidson et al., 2016). Fourteen-day-old fungal plate cultures were flooded with sterile water and pycnidiospores were harvested by gently disturbing the surface with a sterile glass rod. Spore suspensions were filtered through a 250 mm sieve to separate the spores from mycelia and the resultant concentration adjusted to 1 × 10^6^ spores/mL using a haemocytometer. Two to three drops of Tween 20 (0.02% v/v) per 100 mL of spore suspension was added as a surfactant. Subsequently, 3-week-old seedlings of each wild *Lens* genotype and 2 week old seedlings of both controls were uniformly inoculated using an air pressurized hand sprayer until run off. Control/non-inoculated pots were sprayed with water mixed with Tween 20 (0.02% v/v).

Meanwhile, bioassay conditions were adapted from Chen and Muehlbauer (2003) and Davidson et al. (2016) to stimulate the development of blight symptoms on plants. Post inoculation, all pots were covered with long inverted solid paper cups and placed in plastic crates filled with 2–4 cm of water to facilitate 24 h of leaf wetness and darkness. After 48 h, the cups were removed and the plants were covered with wet hessian bags to maintain high humidity until first appearance of disease symptoms. Further, plants were also misted thrice daily to improve the spore germination of the fungus.

#### Disease assessment

Each of the three seedlings per pot was scored for symptoms of *A. lentis* infection at 14 and 21 days post inoculation (dpi) (Ford et al., 1999; Sambasivam et al., 2017) using a non-destructive 1–9 scoring scale specifying a size limit on leaf and stem lesions and percentage leaf drop (Ford et al., 1999; Davidson et al., 2016; Sambasivam et al., 2017). The scores were 1 = no disease symptoms; 3 = leaf lesions only, chlorosis of affected leaves, <10% leaf drop; 5 = leaf lesions, up to 25% leaf drop, stem flecks, or lesions <2 mm; 7 = leaf lesions, up to 50% leaf drop, stem flecks or lesions >2 mm; 9 = leaf lesions, potential defoliation, stem girdling and potential plant death (adapted from Davidson et al., 2016).

#### Statistical analysis

Statistical analysis was performed using IBM SPSS statistics software. Data from all the control (non-inoculated) replicates were excluded from analysis since plants were symptom free with a consistent score of 1. Modes of disease scores of each pot were calculated to study each (genotype × isolate) interaction and Friedman's non-parametric analysis of variance was used to assess the modal variances among them. Most frequently observed scores pooled from three inoculated replicates were used to calculate modal disease score at 14 and 21 dpi. Modal disease scores were used to categorize the genotypes into resistant (1–3), moderately resistant (5) and susceptible (7–9) (Ford et al., 1999; Nguyen et al., 2001; Rubeena et al., 2006). Area under disease progress curve (AUDPC) was used to summarize the disease intensity over time and was estimated as described by Campbell and Madden (1990).

(1) $$\begin{array}{l} \text{AUDPC}=\overset{n}{\underset{i=1}{\sum }}\left(\frac{y_{i}+\;y_{i+1}}{2}\right)\left(t_{i+1}-\;t_{i}\right) \end{array}$$

Where; n = total number of observations, *y*_*i*_ = modal disease score at the *i*th observation and *t* = time at the *i*th observation.

## Results

### Phenotyping of wild genotype resistance to two most aggressive isolates of *A. lentis*

From preliminary screening on ILL 7537, Nipper and ILL 6002, both isolates FT13038 and FT13037 were deemed aggressive, producing a susceptible reaction on ILL 6002 and Nipper with extensive leaf lesions, stem girdling and subsequent plant death at 21 dpi. Further, both isolates produced leaf lesions on ILL7537, with isolate FT13037 (Modal disease score of 7) more aggressive than isolate FT13038 (Modal disease score of 3) (*P* = *0.001*) (Table 2).

**Table 2:** Modal disease score of three host differentials at 14 and 21 dpi for *A. lentis* isolates FT13038 and FT13037.

**S. No**	**Isolate/Genotype**	**FT13037**		**FT13038**	
		**14 DPI**	**21 DPI**	**14 DPI**	**21 DPI**
1	ILL 6002	7	9	7	9
2	ILL 7537	5	7	3	3
3	Nipper	7	9	7	9

Following inoculation of the 30 wild *Lens* genotypes with these isolates, first visual symptoms (leaf lesions) occurred from 7 dpi and stem lesions coalesced leading to stem girdling and plant death by 21 dpi on the most susceptible genotypes. Disease symptoms did not appear until 11 dpi on other genotypes and several were observed to overcome the infection. This demonstrated a range of disease reactions, from susceptible to resistant based on Friedman's test (*P* = 0.002). Two genotypes of *L. orientalis*, ILWL 180 and ILWL 7, were resistant to both isolates at 21 dpi with modal disease scores of 1 and 3, respectively, whereas five genotypes of *L. nigricans*, ILWL 37, PI 572348, PI 572351, PI 572359, and PI 615677, were resistant to just isolate FT13038 (Table 3).

**Table 3:** Details of the genotypes used in the study along with corresponding modal disease scores at 14 and 21 DPI and AUDPC.

**S. No**	**Isolate/Genotype**	**Species**	**Country**	**FT13038**				**FT13037**			
				**14 dpi**	**21 dpi**	**AUDPC**	**Category**	**14 dpi**	**21 dpi**	**AUDPC**	**Category**
1	ILL 6002^a^	*culinaris*	A pure line selection from Argentinian variety, Precoz	5	9	79.94	S	7	9	100.38	S
2	ILL 7537^b^	*culinaris*	Jordan	3	3	46.66	R	5	7	71.75	S
3	PI 572330	*ervoides*	Israel	5	7	74.69	S	7	9	107.1	S
4	PI 572317	*ervoides*	Italy	5	7	73.89	S	7	9	106.54	S
5	PI 572362	*odomensis*	Unknown	5	5	76.23	MR	9	9	123.66	S
6	PI 572336	*ervoides*	Turkey	5	7	79.35	S	5	9	84	S
7	ILWL 172	*odomensis*	Syria	3	7	57.54	S	9	9	120.54	S
8	ILWL 206	*ervoides*	Bosnia and Herzegovina	7	7	90.41	S	7	9	105.14	S
9	ILWL 116	*odomensis*	Syria	5	5	72.35	MR	7	9	105.77	S
10	PI 572342	*nigricans*	France	5	9	82.81	S	7	9	100.28	S
12	ILWL 221	*odomensis*	Turkey	5	5	75.46	MR	5	9	77.77	S
11	ILWL 235	*odomensis*	Syria	5	5	73.89	MR	5	7	80.12	S
14	PI 572345	*nigricans*	Italy	5	5	72.35	MR	5	7	77.77	S
16	PI 572334	*ervoides*	Turkey	5	5	68.46	MR	7	7	87.89	S
17	ILWL 261	*ervoides*	Turkey	5	5	70.81	MR	5	7	75.43	S
19	ILWL 325	*orientalis*	Jordan	5	5	70	MR	5	7	75.81	S
21	ILWL 69	*orientalis*	Former Soviet Union	3	5	52.12	MR	5	7	75.08	S
25	ILWL 70	*orientalis*	Iran	5	7	77	S	5	7	77	S
13	ILWL 146	*orientalis*	Syria	3	5	49	MR	5	5	70	MR
15	PI 572360	*odomensis*	Israel	5	5	66.12	MR	5	5	74.66	MR
18	ILWL 437	*lamottei*	Turkey	5	5	71.96	MR	5	5	74.66	MR
20	PI 572399	*orientalis*	Bosnia and Herzegovina	3	7	59.12	S	5	5	73.89	MR
22	ILWL 160	*odomensis*	Syria	5	7	76.97	S	5	5	69.97	MR
23	PI 572348	*nigricans*	Yugoslavia	3	3	45.12	R	3	5	53.69	MR
24	PI 572347	*nigricans*	Italy	5	5	66.89	MR	3	5	52.92	MR
26	PI 572359	*nigricans*	Turkey	3	3	44.31	R	3	5	52.12	MR
27	PI 572333	*ervoides*	Turkey	3	5	49.39	MR	5	5	66.12	MR
28	PI 615677	*nigricans*	Yugoslavia	3	3	45.12	R	3	5	49.77	MR
29	ILWL 37	*nigricans*	Turkey	3	3	48.23	R	3	5	50.58	MR
30	PI 572351	*nigricans*	Bosnia and Herzegovina	3	3	41.23	R	3	5	48.62	MR
31	ILWL 7	*orientalis*	Turkey	3	3	50.58	R	3	3	44.31	R
32	ILWL 180	*orientalis*	Syria	1	1	19.46	R	1	1	18.69	R

*Genotypes are arranged in descending order of overall resistance for FT13037 isolate. Scores 0 = no disease to 9 = plant death*.

a *ILL 6002–susceptible control*.

b *ILL 7537–resistant control*.

Unsurprisingly, disease severity increased significantly between 14 and 21 dpi for most of the genotypes assessed and when inoculated with either isolate. However, disease severity on ILWL 221, ILWL 235, ILWL 261, ILWL 325, PI 572334, PI 572345, PI 572347, PI 572348, and PI 572360, to isolate FT13038 did not progress after 14 dpi, potentially indicating stability of the resistance response(s) to this isolate. These accessions did however become susceptible at 21 dpi following inoculation with isolate FT13037. Similarly, ILL 7537 was resistant to isolate FT13038 but susceptible to isolate FT13037 at 21 dpi (Table 3).

Significant differences were observed among the AUDPC of genotypes following inoculation with either of the highly aggressive isolates. Isolate FT13037 was able to cause significantly more disease on 25 of the genotypes, and on the two controls, compared to isolate FT13038. The remaining five genotypes, ILWL 70, ILWL 160, PI 572347, ILWL 7, and ILWL 180, had equal or significantly higher disease over time when inoculated with isolate FT13038 compared to isolate FT13037. The highest and lowest disease severity and AUDPC was observed on genotypes ILWL 206 (90.41) and ILWL 180 (19.46), respectively when inoculated with isolate FT13038. Meanwhile, the highest and lowest disease severity and AUDPC was observed on genotypes PI 572362 (123.66) and ILWL 180 (18.69), respectively when inoculated with isolate FT13037.

Five genotypes, PI 572348, PI 572359, PI 615677 PI 572351, and ILWL 180, were more resistant than ILL7537 to isolate FT13038, and 11 genotypes, ILWLW 146, ILWL 160, PI 572333, PI 572348, PI 572347, PI 572359, ILWL 37, PI 615677, PI 572351, ILWL 7, and ILWL 180, were more resistant than ILL7537 to isolate FT13037. Likewise, two genotypes, ILWL 206 and PI 572342, were more susceptible than ILL6002 to isolate FT13038, and six genotypes, PI 572362, ILWL 172, PI 572330, PI 572317, ILWL 116, and ILWL 206, were more susceptible than ILL6002 to isolate FT13037 (Table 3).

### *Lens orientalis* ILWL 180 as a potential novel resistance source

**Figure 1:**
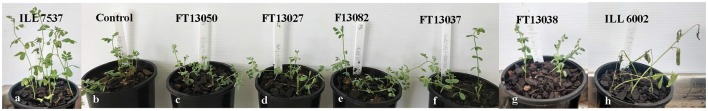
Response of wild *Lens* ILWL 180 to five isolates. **(a,h)** Response of resistant and susceptible controls ILL 7537 and ILL 6002, respectively to a representative isolate FT13038 21 days post inoculation. **(b)** control pot of wild *Lens* ILWL 180. **(c–g)** Response of wild *Lens* ILWL 180 to isolates FT13050, FT13027, F13082, FT13037 and FT13038 21 days post inoculation

The genotype ILWL 180 remained resistant at 21 dpi following repeated screening with the initial two isolates as well as three further isolates FT13027, FT13050, and FT13082 (*P* = *0.534*). This remained so even against what appeared to be the most aggressive isolate FT13037, which was able to overcome ILL7537 (*P* = *0.001*) (Figure 1; Table 4).

**Table 4:** Modal disease scores of ILWL 180 and controls at 14 and 21 dpi against five *A. lentis* isolates

**S. No**	**Isolates**	**ILL 6002**		**ILWL 180**		**ILL 7537**	
		**14 dpi**	**21 dpi**	**14 dpi**	**21DP1**	**14 dpi**	**21 dpi**
1	FT13037^a^	7	7	3	3	5	7
2	FT13038^a^	5	7	1	3	3	3
3	FT13050^a^	7	7	1	3	3	3
4	FT13027^a^	7	7	3	3	3	3
5	FT13082^b^	5	5	1	1	1	3

a *aggressive*.

b *non-aggressive*.

## Discussion

Evolution of the pathogen population toward more highly aggressive isolates has likely contributed to failure and reclassification of the resistance status of widely grown cultivars, such as Laird and breeding line ILL 5588 in Canada (Morrall, 1997; Morrall et al., 2004), and Northfield and Nipper in Australia (Nasir and Bretag, 1997b; Davidson et al., 2016), although this requires further spatial and temporal population assessment for validation. Furthermore, the broad diversity within the *A. lentis* population will likely maintain pressure on the few remaining resistance sources within the Australian cultivars (Davidson et al., 2016). Hence, introduction of potentially novel resistance sources from diverse germplasm, such as wild relatives is pivotal for maintaining production stability within the lentil industry.

The recent inclusion of ILL7537 as a resistance source within the Australian breeding program was largely consistent with the findings of this study, whereby this accession was resistant against the majority of isolates assessed. Although none of the existing varieties have ILL7537 as one of the parent in their pedigree, isolate FT13037, which was isolated in 2013 from Urania, the Yorke Peninsula of South Australia, was able to cause severe disease on ILL7537 under the bioassay conditions. Therefore, caution should be taken when relying upon this source of resistance for future resistance breeding strategies. The resistance status of this source and Indianhead was previously questioned following controlled bioassays (Nguyen et al., 2001; Davidson et al., 2016).

The quantitative summary of disease severity and progression in this study identified resistant genotypes from *L. orientalis* (2) and *L. nigricans* (5) but not from *L. odomensis, L. ervoides* or *L. lamottei*. This agreed with the findings of Tullu et al. (2010), who reported ILWL 206 (*L. ervoides*) as susceptible and ILWL 146 (*L. orientalis*) as moderately resistant against Canadian isolates. However, this was in contrast to the previous findings of Bayaa et al. (1994), who reported that ILWL 69, ILWL 116, ILWL 172, ILWL 206, and ILWL 261 were resistant to Syrian isolates, potentially indicating a higher aggressiveness of isolates within the current Australian population. The two *L. orientalis* genotypes identified in this study as resistant (ILWL 180 and ILWL 7) and moderately resistant (ILWL 146) were previously also reported to be resistant to Syrian isolates (Bayaa et al., 1994), potentially highlighting the stability of these resistance sources. Similarly, Tullu et al. (2010) identified *L. ervoides, L. nigricans*, and *L. orientalis* genotypes resistant to both Canadian and Syrian isolates, also highlighting that the wild species may possess broad resistances.

Interestingly, the resistant genotypes ILWL 180 and ILWL 146 originated from a common geographical region of Syria and other moderately resistant genotypes originated from Turkey (Bayaa et al., 1994). Associations between geographical origin and the *A. lentis* resistance trait have previously been reported in larger germplasm collections representative of different geographical regions (Bayaa et al., 1994), indicating potential co-evolution of resistance mechanisms with selection from regional populations. Given that these accessions are also resistant to the most aggressive Australian isolates, shared environmental-trait (resistance) based relationships would be useful to consider when seeking further resistance sources within germplasm collections. For this, researchers at the International Centre for Agricultural Research in Dry Areas (ICARDA) have developed a Focused Identification of Germplasm Strategy (FIGS) (Mackay, 1990, 1995, 2011; Street et al., 2008).

After identification in a wild relative species (subspecies), the next hurdle is to bring the desirable genes/alleles across to an elite cultivated background. For *Lens*, inter-species crossing has been encumbered with pre- and post-fertilization barriers, such as reduced pollen fertility, chromosomal aberrations and embryo abortion (Abbo and Ladizinsky, 1991, 1994; Gupta and Sharma, 2007). To date, no successful deployment of wild relative-derived resistance for improved *A. lentis* resistance has been reported. Nevertheless, fertile and phenotypically normal hybrids have been created between primary gene pool species, such as *L. culinaris* and *L. orientalis* through conventional techniques (Wong et al., 2015). Thus, exploiting the resistance detected in *L. orientalis* would be a practical choice rather than pursuing that detected in secondary, tertiary or quaternary gene pools, which would be time consuming and laborious.

In conclusion, substantial variation for resistance to *A. lentis* is present in wild relative genepools and the *L. orientalis* accession ILWL 180 was most resistant to the most highly aggressive isolates detected in the recent Australian population. Further investigation into this resistance source is required to validate its stability against the breadth of the pathogen population and to identify resistance loci for selective breeding purposes.

## Author contributions

RD conducted bioassays, wrote the manuscript and analyzed the data. DG, RF, and PS conceived the study, participated in its design and assisted to draft the manuscript. All authors read and approved the final manuscript.

### Conflict of interest statement

The authors declare that the research was conducted in the absence of any commercial or financial relationships that could be construed as a potential conflict of interest.
